# Improved liver function in patients with cirrhosis due to chronic hepatitis C virus who achieve sustained virologic response is not accompanied by increased liver volume

**DOI:** 10.1371/journal.pone.0231836

**Published:** 2020-04-20

**Authors:** Taijiro Wake, Ryosuke Tateishi, Tsuyoshi Fukumoto, Ryo Nakagomi, Mizuki Nishibatake Kinoshita, Takuma Nakatsuka, Masaya Sato, Tatsuya Minami, Koji Uchino, Kenichiro Enooku, Hayato Nakagawa, Hidetaka Fujinaga, Yoshinari Asaoka, Yasuo Tanaka, Motoyuki Otsuka, Kazuhiko Koike

**Affiliations:** 1 Department of Gastroenterology, Graduate School of Medicine, The University of Tokyo, Tokyo, Japan; 2 Department of Gastroenterology, Teikyo University, Tokyo, Japan; Nihon University School of Medicine, JAPAN

## Abstract

**Background:**

Serum albumin level improves in patients with chronic hepatitis C virus (HCV) infection who achieve sustained virologic response (SVR) with antiviral therapy. However, it remains controversial whether liver volume increases along with SVR.

**Methods:**

Patients with chronic HCV infection with a history of hepatocellular carcinoma (HCC) who achieved SVR with anti-HCV treatment from March 2003 to November 2017 were enrolled. Patients were followed up with periodic computed tomography (CT) scans to detect HCC recurrence. Patients who underwent treatment for HCC recurrence within 1 year after initiation of anti-HCV treatment were excluded. Laboratory data, including alanine aminotransferase (ALT) level, serum albumin level, and platelet count, were collected at baseline and timepoints after treatment initiation. Liver volume was evaluated at baseline and 24 and 48 weeks after treatment initiation using a CT volume analyzer. A linear mixed-effects model was applied to analyze the chronologic change in liver volume. The correlations between changes in ALT level, albumin level, and liver volume were also evaluated.

**Results:**

Of 108 enrolled patients, 78 had cirrhosis. Serum albumin level continued to increase through 48 weeks after treatment initiation. A significant increase in liver volume was observed only in patients without cirrhosis (*P* = 0.005). There was a significant correlation between ALT level decrease and albumin level increase (*P* = 0.018).

**Conclusions:**

Improved liver albumin production with SVR was contributed by improved liver cell function rather than increased liver volume in patients with cirrhosis.

## Introduction

More than 180 million people worldwide are infected with hepatitis C virus (HCV) [1]. HCV is a major cause of chronic liver disease, including cirrhosis and hepatocellular carcinoma (HCC) [2, 3]. Interferon (IFN) therapy has been proved to achieve sustained virologic response (SVR) in patients with chronic HCV infection and can prevent progression of liver fibrosis. Some studies have also suggested that IFN therapy can reduce the risk of HCC [4–6]. However, until recently, IFN-containing regimens have shown unsatisfactory SVR rates in patients with advanced fibrosis or cirrhosis due to lower dose intensity and adherence as a result of more severe adverse effects in these patients [7–9]. Currently, IFN-free, all-oral regimens with direct-acting antivirals (DAAs) have shown nearly 100% SVR rates in patients not suitable for IFN-containing regimens [10–12].

Liver volume increases or decreases according to the clinical course of various liver diseases. For example, marked atrophy is observed in acute liver failure [13–15], whereas hepatomegaly is prevalent in alcoholic hepatitis [16, 17]. In general, liver volume decreases in advanced cirrhosis, a final form of liver fibrosis as a consequence of long lasting liver damage and scarring [18]. On the other hand, previous studies reported improved liver fibrosis in patients with CHC after SVR [19, 20]. Recently, it is reported that liver volume increased in patients with CHC who achieved SVR with IFN-based therapy [21]. However, most patients included in the study were non-cirrhotic since advanced cirrhosis are not indicated for IFN-based therapy. The aim of this study is clarify the relationship between improved liver function and chronologic change in liver volume in patients with chronic HCV infection who achieved SVR with anti-HCV treatment.

## Patients and methods

### Patient enrollment

This retrospective study was conducted according to the ethical guidelines for epidemiologic research of the Japanese Ministry of Education, Culture, Sports, Science and Technology, and the Ministry of Health, Labor and Welfare. This study was included in a comprehensive protocol of retrospective studies in the Department of Gastroenterology of the University of Tokyo Hospital and was approved by the University of Tokyo Medical Research Center Ethics Committee (approval number 2058). Informed consent was waived because of the retrospective design. The following statements were posted at a website (http://gastro.m.u-tokyo.ac.jp/patient/clinicalresearch.html) and participants who do not agree to the use of their clinical data can claim deletion of them.

Department of Gastroenterology at The University of Tokyo Hospital contains data from our daily practice for the assessment of short-term (treatment success, immediate adverse events etc.) and long-term (late complications, recurrence etc.) outcomes. Obtained data were stored in an encrypted hard disk separated from outside of the hospital. When reporting analyzed data, we protect the anonymity of participants for the sake of privacy protection. If you do not wish the utilization of your data for the clinical study or have any question on the research content, please do not hesitate to make contact with us.

**Fig 1:**
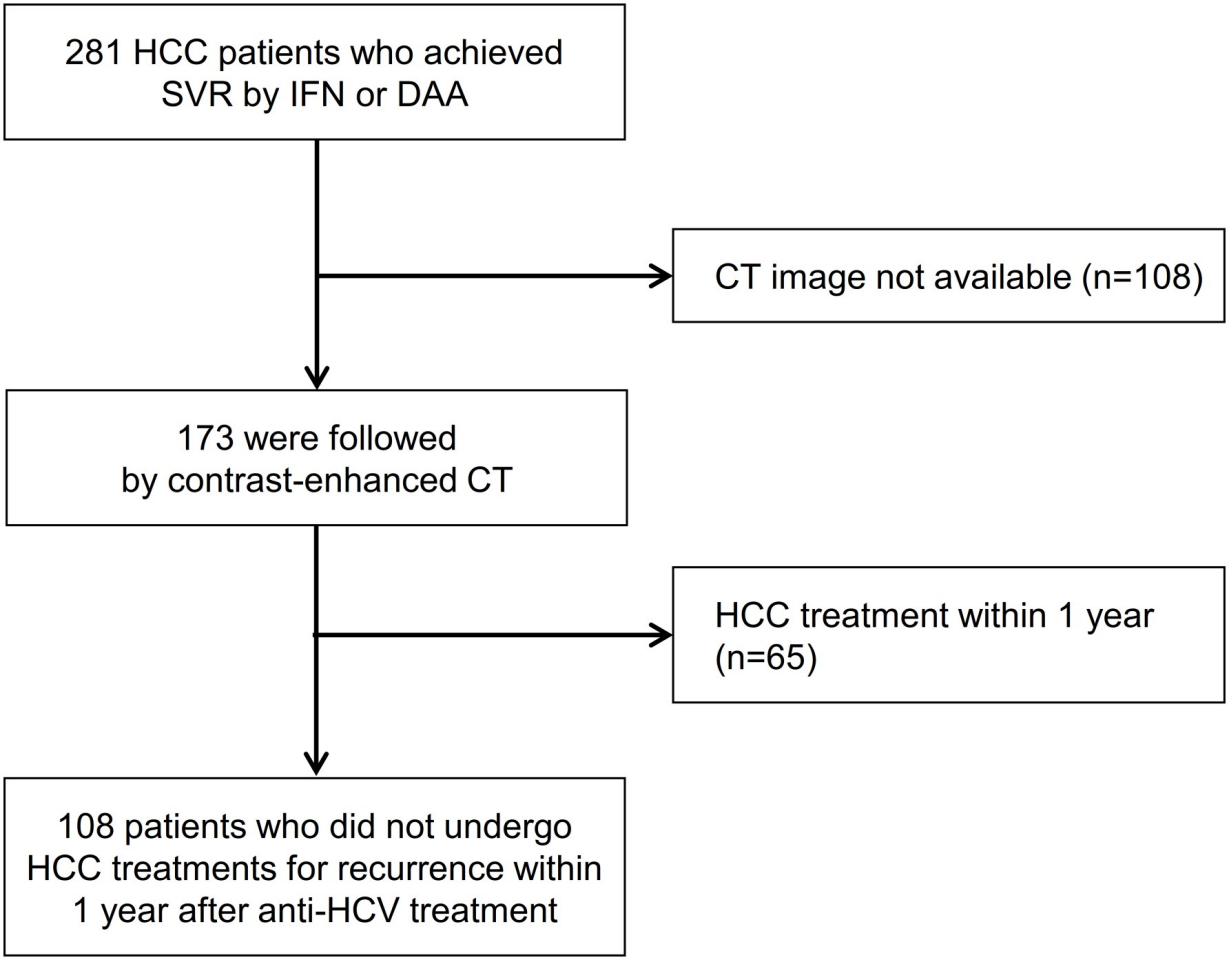
Patient flow diagram. CT, computed tomography; DAA, direct-acting antiviral; HCC, hepatocellular carcinoma; HCV, hepatitis C virus; IFN, interferon; SVR, sustained virologic response.

Patients with chronic HCV infection with a history of HCC who achieved SVR with IFN-based or IFN-free DAA therapy from March 2003 to November 2017 were enrolled. SVR was defined as no detectable virus on quantitative RNA testing at 24 weeks posttreatment. Treatment duration was 24 or 48 weeks for IFN therapy and 12 or 24 weeks for DAA therapy. Patients were followed up with periodic computed tomography (CT) scans to detect HCC recurrence. Patients who were followed up with magnetic resonance imaging or who underwent treatment for HCC recurrence within 1 year after initiation of anti-HCV treatment were excluded (Fig 1). Thus, no viable HCC nodules were detected in all enrolled patients during the study period. Patients for whom enhanced CT images were not available for all 3 timepoints—within 16 weeks before treatment and at 24 and 48 weeks after treatment initiation—were also excluded. Patients were diagnosed with cirrhosis based on the following criteria: METAVIR stage 4 fibrosis by liver biopsy [22], presence of gastroesophageal varices, platelet count <10^9^/μL with splenomegaly, or typical morphological change in liver imaging with deteriorated liver function.

### Data collection

Baseline characteristics of enrolled patients, including age, sex, obesity (defined as body mass index > 25 kg/m^2^), history of alcohol consumption, liver cirrhosis, diabetes mellitus, albumin, total bilirubin, aspartate aminotransferase, alanine aminotransferase (ALT), γ-glutamyltransferase, prothrombin time, platelet count, creatinine, liver volume, and Fibrosis-4 index (FIB-4) were obtained before anti-HCV treatment [23]. The chronologic change in albumin level was evaluated at baseline and 12, 24, and 48 weeks after treatment initiation. ALT level was evaluated at baseline and 4, 8, and 12 weeks after treatment initiation. Serum bilirubin level and platelet count were evaluated at baseline and 24 and 48 weeks after treatment initiation.

### Liver volume measurement

Liver volume was evaluated at baseline and 24 and 48 weeks after treatment initiation using a CT volume analyzer (Synapse Vincent; Fujifilm Corp, Tokyo, Japan) [24]. Digital Imaging and Communication in Medicine–formatted CT data were transferred from a server to a workstation and liver volume was measured in the delayed phase of dynamic CT scans; nonenhanced areas (e.g., cyst, previously ablated area, lipiodol deposition) were excluded. Liver volume was standardized based on estimated liver volume calculated using the following formula [14]:

$$\text{standard}\;\text{liver}\;\text{volume}\;({cm}^{3})=706.2\times \text{body}\;\text{surface}\;\text{area}\;(m^{2})+2.4.$$

Liver volume was measured by 1 trained hepatologist (TW). To evaluate the reproducibility of liver volume measurement, 10 subjects from this cohort were randomly selected and liver volume at each timepoint was measured by another trained hepatologist (TF).

Additionally, we evaluated spleen volume at baseline and the presence of collateral circulation in cirrhotic patients.

### Statistical analysis

Data are presented as mean and standard deviation or median and interquartile range (IQR) for quantitative variables, and as number and percentage for qualitative variables. Liver volume was assessed as a proportion of total functional liver volume before anti-HCV treatment. The reproducibility of liver volume measurement was assessed by comparing the data from the 2 independent observers and reported as the correlation coefficient.

A linear mixed-effects model was applied to analyze the chronologic change in outcome variables including liver volume, albumin level, platelet count, and total bilirubin level. We included age, sex, albumin level, and ALT level at baseline in the fixed part of the model and added a random-effect intercept for each subject to account for correlations among repeated measurements per person. Subgroup analysis was also performed, dividing the whole cohort into patients with and without cirrhosis and patients with a Child-Pugh score of 5 and 6. The cirrhotic patients were further divided into two groups based on the presence of collateral circulation. The following correlations were evaluated using scatter plots and the Spearman correlation coefficient: albumin level, FIB-4 index and liver volume at baseline and change in ALT level, albumin level, and liver volume. We also evaluated the correlation between platelet count and spleen volume.

All statistical analyses were performed using R version 3.2.3 (R Development Core Team, Vienna, Austria) with *P* values < 0.05 considered to be significant.

## Results

### Patient profiles

Baseline characteristics of the patients are shown in Table 1. The mean age was 69 years (range, 40–87 years) and patients were predominantly male (68%). Seventy-eight patients (72.2%) had cirrhosis. The median liver volume at baseline was 1238 cm^3^ (IQR, 1033–1439 cm^3^). Collateral circulation was observed in 38 (48.7%) of 78 cirrhotic patients. Spleen volume was measured in 73 cirrhotic patients; the remaining 5 patients had undergone splenectomy. The median (IQR) of spleen volume was 197 (152–292) cm^3^.

**Table 1:** Baseline characteristics (N = 108)*.

Characteristic	N = 108
Age, y	
Mean ± SD	69 ± 8.6
Range	40 -87
Male sex, n (%)	74 (68)
BMI > 25 kg/m^2^, n (%)	27 (25)
Alcohol intake > 80 g/d, n (%)	7 (6.5)
Liver cirrhosis, n (%)	78 (72)
Child-Pugh score 5, n (%)	78 (72)
Diabetes mellitus, n (%)	22 (20)
Albumin, g/dL	3.8 (3.5–4.0)
Total bilirubin, mg/dL	0.8 (0.7–1.0)
AST, U/L	57 (41–76)
ALT, U/L	57 (34–80)
GGT, U/L	42 (27–64)
Platelet count, × 10^4^/μL	11.5 (8.8–15.6)
Creatinine, mg/dL	0.74 (0.65–0.85)
Liver volume, cm^3^	1238 (1033–1439)
FIB-4 index	4.89 (3.02–7.42)
Treatment regimen, n (%)	
IFNα	1 (0.9)
PEG-IFN	8 (7.4)
PEG-IFN/RBV	30 (27.8)
PEG-IFN/RBV/DAA	2 (1.9)
DCV/ASV	19 (17.6)
SOF/RBV	11 (10.2)
SOF/LDV	33 (30.5)
EBR/GZR	4 (3.7)

* Values are expressed as median (25th-75th percentiles) or n (%).

ALT, alanine aminotransferase; AST, aspartate aminotransferase; ASV, asunaprevir; BMI, body mass index; DAA, direct-acting antiviral; DCV, daclatasvir; EBR, elbasvir; FIB, fibrosis; GGT, ɤ-glutamyltransferase; GZR, grazoprevir; IFN, interferon; LDV, ledipasvir; PEG-IFN, pegylated interferon; RBV, ribavirin; SOF, sofosbuvir.

### Correlation of baseline variables

There was a significant inverse correlation between platelet count and spleen volume adjusted by body surface (S1 Fig) and albumin level and liver volume at baseline (S2A Fig). On the other hand, there was no significant correlation between FIB-4 and liver volume (S2B Fig).

### Chronologic changes in laboratory data

**Fig 2:**
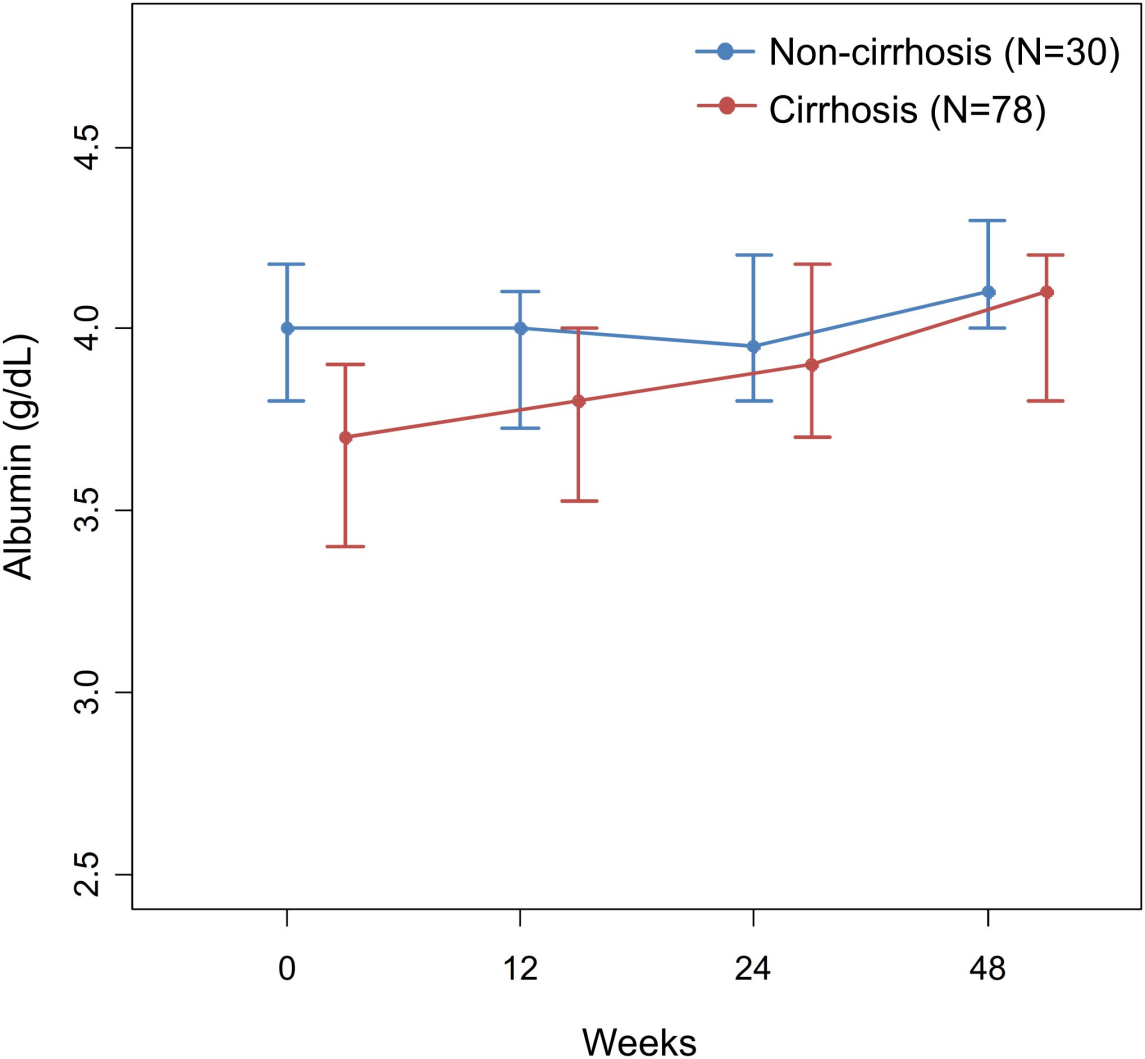
Chronologic changes in serum albumin (A) and ALT levels (B) in non-cirrhosis (blue line) vs cirrhosis (red line). Points and error bars indicate median values and 25th to 75th percentiles, respectively. ALT, alanine aminotransferase.

Fig 2 shows the chronologic changes in serum albumin and ALT levels. The serum albumin level continued to increase through 48 weeks after treatment initiation (*P* = 0.004 and <0.001, respectively by linear mixed effect model). The ALT level decreased rapidly in both groups at 4 weeks after treatment initiation (*P* <0.001 and <0.001, respectively by the Wilcoxon rank-sum test). In total, albumin level increased in 84 patients (78%) at 48 weeks, while ALT level decreased in 95 patients (88%) at 12 weeks. The chronologic changes in platelet count and total bilirubin level are shown in S3 Fig. Platelet count significantly increased in both groups (*P* = 0.035 and <0.001 by linear mixed effect model) whereas total bilirubin did not change significantly.

### Change in liver volume

**Fig 3:**
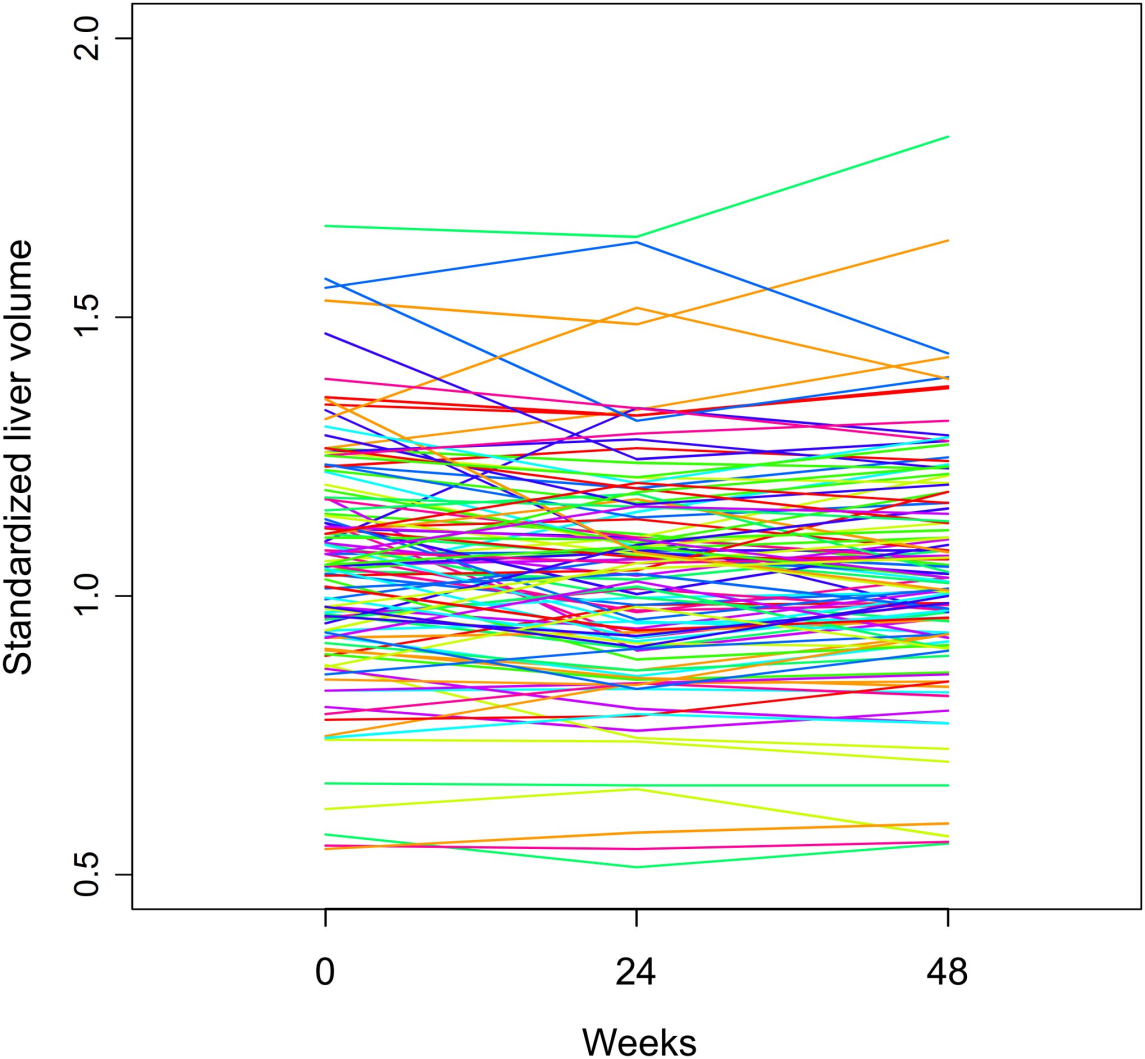
Chronologic change in standardized liver volume from baseline to 24 and 48 weeks after treatment initiation. (A) Each line indicates a unique patient. (B) Points and error bars indicate median values and 25th to 75th percentiles, respectively.

Measurement of liver volume was consistent and highly reproducible among observers (correlation coefficient = 0.99; S4 Fig). Fig 3 shows the chronologic change in liver volume from enrollment to 24 and 48 weeks after treatment initiation. The median (IQR) values of standardized liver volume at baseline and 24 and 48 weeks after treatment initiation were 1.057 (0.937–1.180), 1.054 (0.914–1.159), and 1.032 (0.932–1.167), respectively. Compared with baseline values, liver volume increased in 16 of 30 patients without cirrhosis (53%) and in 29 of 78 patients with cirrhosis (37%) at 48 weeks (S5 Fig).

A linear mixed-effects model showed that there was no significant increase in liver volume after treatment initiation (Table 2). However, subgroup analysis showed that there was a significant increase in liver volume in patients without cirrhosis (Table 3).

**Table 2:** Linear mixed-effects model of liver volume (N = 108)*.

Variable	Regression Coefficient	95% CI	*P* value
Age, per year	-0.007	-0.096 to -0.082	0.002
Female vs male	-0.034	-0.11–0.043	0.38
Albumin, per 1.0 g/dL	0.146	0.059–0.233	0.001
ALT, per 1.0 U/L	0.001	0.0004–0.002	0.003
Liver volume measurement interval, per 48-week	0.016	-0.009–0.04	0.20

* All data were obtained before HCV treatment. Liver volume was standardized based on estimated total liver volume.

ALT, alanine aminotransferase; HCV, hepatitis C virus.

**Table 3:** Linear mixed-effects model of liver volume in non-cirrhosis vs cirrhosis*.

Variable	Non-cirrhosis (n = 30)			Cirrhosis (n = 78)		
	Coefficient	95% CI	*P* value	Coefficient	95% CI	*P* value
Age, per year	-0.01	-0.023–0.003	0.13	-0.006	-0.01 to -0.002	0.008
Female vs male	-0.06	-0.28–0.16	0.58	-0.026	-0.11–0.06	0.53
Albumin, per 1.0 g/dL	0.26	-0.08–0.61	0.13	0.13	0.03–0.23	0.009
ALT, per 1.0 U/L	0.0013	-0.002 to -0.004	0.37	0.001	0.0004–0.002	0.003
Liver volume measurement interval, per 48-week	0.06	0.0004–0.002	0.005	-0.003	-0.03–0.025	0.85

* All data were obtained before HCV treatment. Liver volume was standardized based on estimated total liver volume.

ALT, alanine aminotransferase; HCV, hepatitis C virus.

The chronologic change in liver volume was not significant in cirrhotic patients. Subgroup analysis with Child-Pugh score also did not show a significant increase in liver volume (S1 Table). We further divided the cirrhotic patients based on the presence of collateral circulation. The results also showed no significant change (S2 Table).

### Correlation between liver function and volume

**Fig 4:**
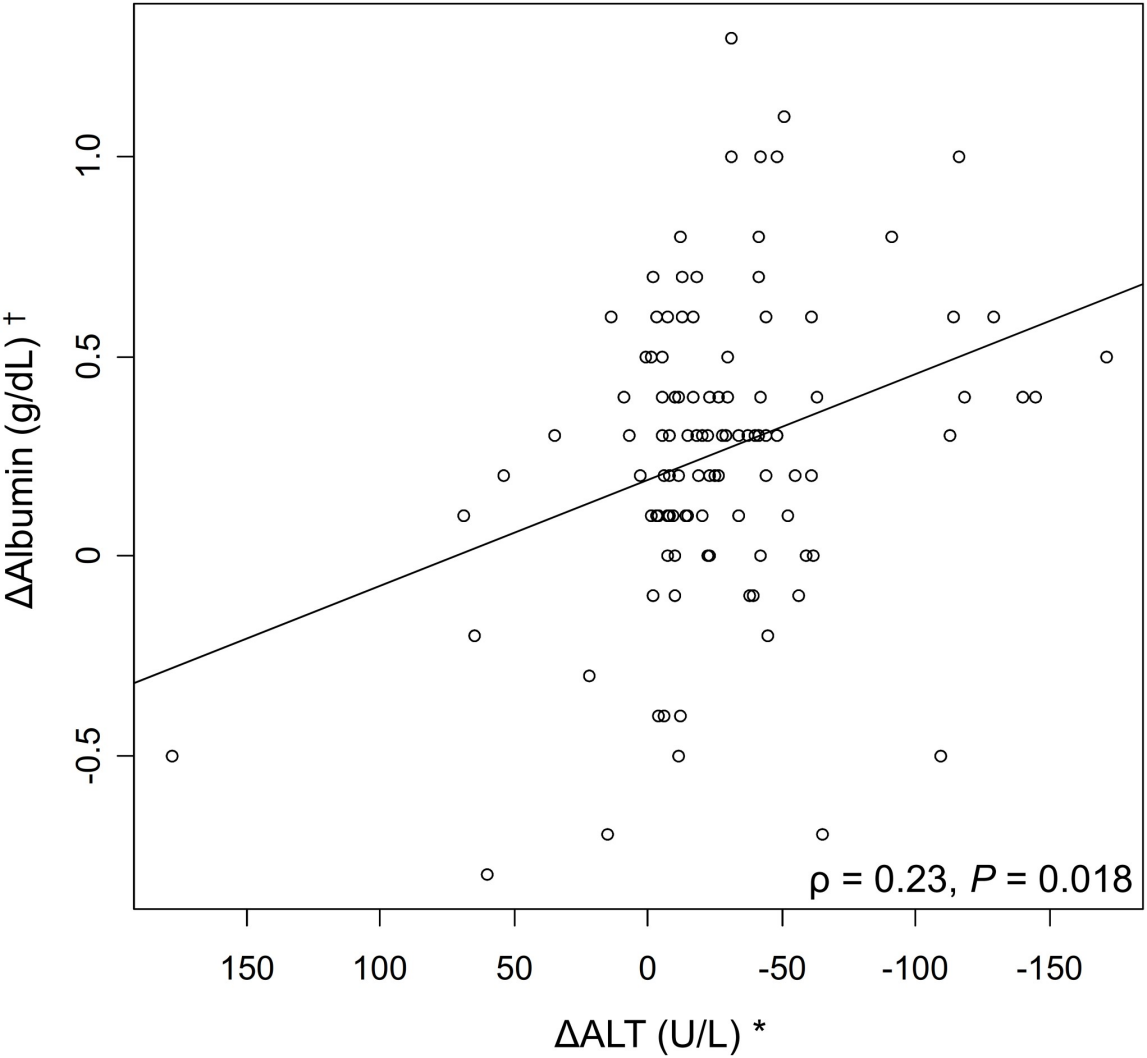
(A) Correlation between ALT and albumin levels.* ΔALT = ALT level at 12 weeks—ALT level at baseline. ^†^ ΔAlbumin = albumin level at 48 weeks—albumin level at baseline. (B) Correlation between albumin level and standardized liver volume.* ΔAlbumin = albumin level at 48 weeks—albumin level at baseline. ^†^ ΔStandardized liver volume (SdLV) = SdLV at 48 weeks—SdLV at baseline. (C) Correlation between ALT level and standardized liver volume.* ΔALT = ALT level at 12 weeks—ALT level at baseline. ^†^ ΔStandardized liver volume (SdLV) = SdLV at 48 weeks—SdLV at baseline. ALT, alanine aminotransferase.

Fig 4 shows the relationships between changes in ALT level, albumin level, and standardized liver volume. There was a significant correlation between decrease in ALT level and increase in albumin level (*P* = 0.018), whereas there were no significant correlations between change in liver volume and changes in albumin and ALT levels (*P* = 0.37 and 0.89, respectively).

## Discussion

Until recently, a limited proportion of patients with cirrhosis could expect benefit from anti-HCV treatment, as pegylated IFN + ribavirin, a former standard regimen, is not indicated for advanced cirrhosis. Even if indicated, the SVR rate is low due to insufficient dose intensity as a result of low platelet count and hemoglobin level in patients with cirrhosis. However, because DAA therapy is indicated for cirrhosis, both patients with and without cirrhosis could be evaluated in this study.

Liver regeneration after liver resection or liver transplantation has been well investigated. After living donor liver transplantation, the volume of the transplanted liver rapidly increases in the first month, then becomes plateau after the volume reaches three fourth of the standard liver volume [25]. The liver regenerates more rapidly in normal liver than diseased liver after liver resection. Yamanaka et al. reported that the rate of liver regeneration after extended hepatectomy was 20 cm^3^/day in normal liver whereas the rate was 11 cm^3^/day in chronic liver disease and 8.5 cm^3^/day in cirrhosis [26]. Ju et al. reported that the remnant liver volume of living liver donor increases by 73%, whereas the increase in liver volume was 55% in cirrhosis after liver resection [27].

CT volume evaluation that enables secure operation plans is now essential before extended hepatectomy and living liver transplantation. CT volume examination had been performed by a hand-tracing method with CT films. Now, we can measure liver volume more rapidly and accurately by using dedicated software and DICOM-formatted digital images.

The liver has natural regenerative ability, and it has been reported that liver function improves after achieving SVR, even in patients with cirrhosis [28]. This study reproduced a previous finding that liver function, as represented by serum albumin level, improved with SVR in patients with and without cirrhosis. On the other hand, liver volume did not increase in patients with cirrhosis. Since it was reported that increases in liver volume after portal vein embolization or hepatic resection were smaller in patients with cirrhosis than in those without cirrhosis [29, 30], it is reasonable that liver volume did not significantly increase in such patients. Therefore, other mechanism should exists behind the improved albumin production in cirrhotic patients. It is well known that albumin level decreases in patients with chronic inflammation [31], and it is reported that interleukin-1 plays a role in decreased liver albumin messenger RNA levels as well as albumin production in a rat model with chronic inflammation [32]. Thus it can be assumed that improved albumin production is a direct consequence of improved liver inflammation after SVR. In fact, there was a significant correlation between increase in serum albumin level and decrease in ALT level with SVR.

Different from the finding in this study, a previous study reported that liver volume increased during anti-HCV treatment in patients who achieved SVR [21]. The reason for this discrepancy may be that 20 of 22 enrolled patients did not have cirrhosis in the previous study, which enrolled patients who received pegylated IFN + ribavirin therapy only [21]. In addition, patients were much younger (median age, 47 years) than those in our study (median age, 67 years).

In long-standing chronic liver disease, the hepatocyte death and regeneration may be balanced. The remission of chronic hepatitis after SVR leads to improvement in hepatocyte function and resolution of fibrosis. Whereas liver regeneration due to the remission in liver inflammation, which occurs within several months after the initiation of anti-HCV treatments, is supposed to occur rapidly, liver fibrosis resolution needs many years. Shiratori et al. reported that it took 3.5 years on average to observe one stage of liver fibrosis resolution after SVR in chronic hepatitis C treated by IFN therapy [19]. Thus a much longer observation period is necessary to observe fibrosis resolution and subsequent liver volume improvement.

This study has several limitations. First, only patients with a history of HCC were enrolled, as it is rare in our hospital to perform periodic CT scans in patients without HCC. Thus, the previous HCC treatment might have hampered the liver’s regenerative ability. However, because albumin levels increased in our cohort with a history of HCC like previous study without HCC, the results can be extrapolated to patients without HCC. Second, approximately 40% of patients were excluded because of HCC recurrence during the observation period. However, because the characteristics were similar between included and excluded patients, the selection bias may not be large.

In conclusion, liver function significantly improved after achieving SVR, even in patients with cirrhosis. However, an increase in liver volume was observed only in patients without cirrhosis.

## Supporting information

S1 Data The clinical data of 108 patients, which were analyzed in this study. (CSV)

S1 Table Linear mixed-effects model of liver volume in Child-Pugh score 5 vs 6. (DOCX)

S2 Table Linear mixed-effects model of liver volume in cirrhosis and collateral circulation (DOCX)

S1 Fig Correlation between platelet count and spleen volume adjusted by body surface at baseline. (TIF)

S2 Fig (A) Correlation between albumin level and liver volume at baseline. (B) Correlation between FIB-4 and liver volume at baseline. FIB-4, Fibrosis-4.
(TIF)

S3 Fig Chronologic changes in platelet count (A) and bilirubin level (B) in non-cirrhosis (blue line) vs cirrhosis (red line). Points and error bars indicate median values and 25th to 75th percentiles, respectively.
(TIF)

S4 Fig Interobserver agreement in liver volume measurement. There was a correlation of 0.99 between values measured by 2 observers.
(TIF)

S5 Fig Chronologic change in standardized liver volume in non-cirrhosis (A) vs cirrhosis (B) from baseline to 24 and 48 weeks after treatment initiation. Each line indicates a unique patient.
(TIF)

## Abbreviations

ALT: alanine aminotransferase
AST: aspartate aminotransferase
ASV: asunaprevir
BMI: body mass index
CHC: chronic hepatitis C
CT: computed tomography
DAA: direct-acting antiviral
DCV: daclatasvir
EBR: elbasvir
FIB: fibrosis
GGT: ɤ-glutamyltransferase
GZR: grazoprevir
HCC: hepatocellular carcinoma
HCV: hepatitis C virus
IFN: interferon
IQR: interquartile range
LDV: ledipasvir
PEG: pegylated
RBV: ribavirin
RFA: radiofrequency ablation
SOF: sofosbuvir
SVR: sustained virologic response
TACE: transcatheter arterial chemoembolization
