# Supplementary material for: Improved liver function in patients with cirrhosis due to chronic hepatitis C virus who achieve sustained virologic response is not accompanied by increased liver volume
Source: PLoS One. 2020 Apr 20;15(4):e0231836. doi: 10.1371/journal.pone.0231836 (PMC7170262; doi:10.1371/journal.pone.0231836)
Supplement: S1 Table — (DOCX) [file pone.0231836.s002.docx]

# Supplementary Table 1. Linear mixed-effects model of liver volume in Child-Pugh score 5 vs 6*

|  | **Child-Pugh score 5 (n = 78)** | | | **Child-Pugh score 6 (n = 30)** | | |
| --- | --- | --- | --- | --- | --- | --- |
| **Variable** | **Coefficient** | **95% CI** | ***P* value** | **Coefficient** | **95% CI** | ***P* value** |
| Age, per year | -0.006 | -0.01 to -0.001 | 0.015 | -0.009 | -0.02–0.0003 | 0.057 |
| Female vs male | -0.068 | -0.15–0.016 | 0.11 | 0.09 | -0.11–0.29 | 0.37 |
| Albumin, per 1.0 g/dL | 0.176 | 0.03–0.32 | 0.017 | 0.21 | -0.03–0.45 | 0.09 |
| ALT, per 1.0 U/L | 0.0015 | 0.0005–0.0025 | 0.002 | 0.0002 | -0.002–0.003 | 0.91 |
| Liver volume measurement interval, per 48-week | 0.021 | -0.008–0.05 | 0.16 | 0.0017 | -0.04–0.05 | 0.94 |

* All data were obtained before HCV treatment. Liver volume was standardized based on estimated total liver volume.

ALT, alanine aminotransferase; HCV, hepatitis C virus.
