# Supplementary material for: Improved liver function in patients with cirrhosis due to chronic hepatitis C virus who achieve sustained virologic response is not accompanied by increased liver volume
Source: PLoS One. 2020 Apr 20;15(4):e0231836. doi: 10.1371/journal.pone.0231836 (PMC7170262; doi:10.1371/journal.pone.0231836)
Supplement: S2 Table — (DOCX) [file pone.0231836.s003.docx]

# Supplementary Table 2. Linear mixed-effects model of liver volume in cirrhosis and collateral circulation*

|  | **Cirrhosis without collateral circulation**  **(n = 40)** | | | **Cirrhosis with collateral circulation**  **(n = 38)** | | |
| --- | --- | --- | --- | --- | --- | --- |
| **Variable** | **Coefficient** | **95% CI** | ***P* value** | **Coefficient** | **95% CI** | ***P* value** |
| Age, per year | -0.009 | -0.014 to -0.004 | <0.001 | -0.005 | -0.01 to -0.002 | 0.19 |
| Female vs male | -0.017 | -0.11–0.07 | 0.71 | -0.016 | -0.15–0.12 | 0.82 |
| Albumin, per 1.0 g/dL | 0.033 | -0.09–0.16 | 0.6 | 0.16 | 0.002–0.32 | 0.048 |
| ALT, per 1.0 U/L | 0.0009 | -0.00004–0.0017 | 0.06 | 0.0018 | 0.0001–0.004 | 0.038 |
| Liver volume measurement interval, per 48-week | 0.015 | -0.023–0.052 | 0.43 | -0.02 | -0.06–0.019 | 0.30 |

* All data were obtained before HCV treatment. Liver volume was standardized based on estimated total liver volume.

ALT, alanine aminotransferase; HCV, hepatitis C virus.
