# Supplementary figures and images for: Improved liver function in patients with cirrhosis due to chronic hepatitis C virus who achieve sustained virologic response is not accompanied by increased liver volume
Source: PLoS One. 2020 Apr 20;15(4):e0231836. doi: 10.1371/journal.pone.0231836 (PMC7170262; doi:10.1371/journal.pone.0231836)

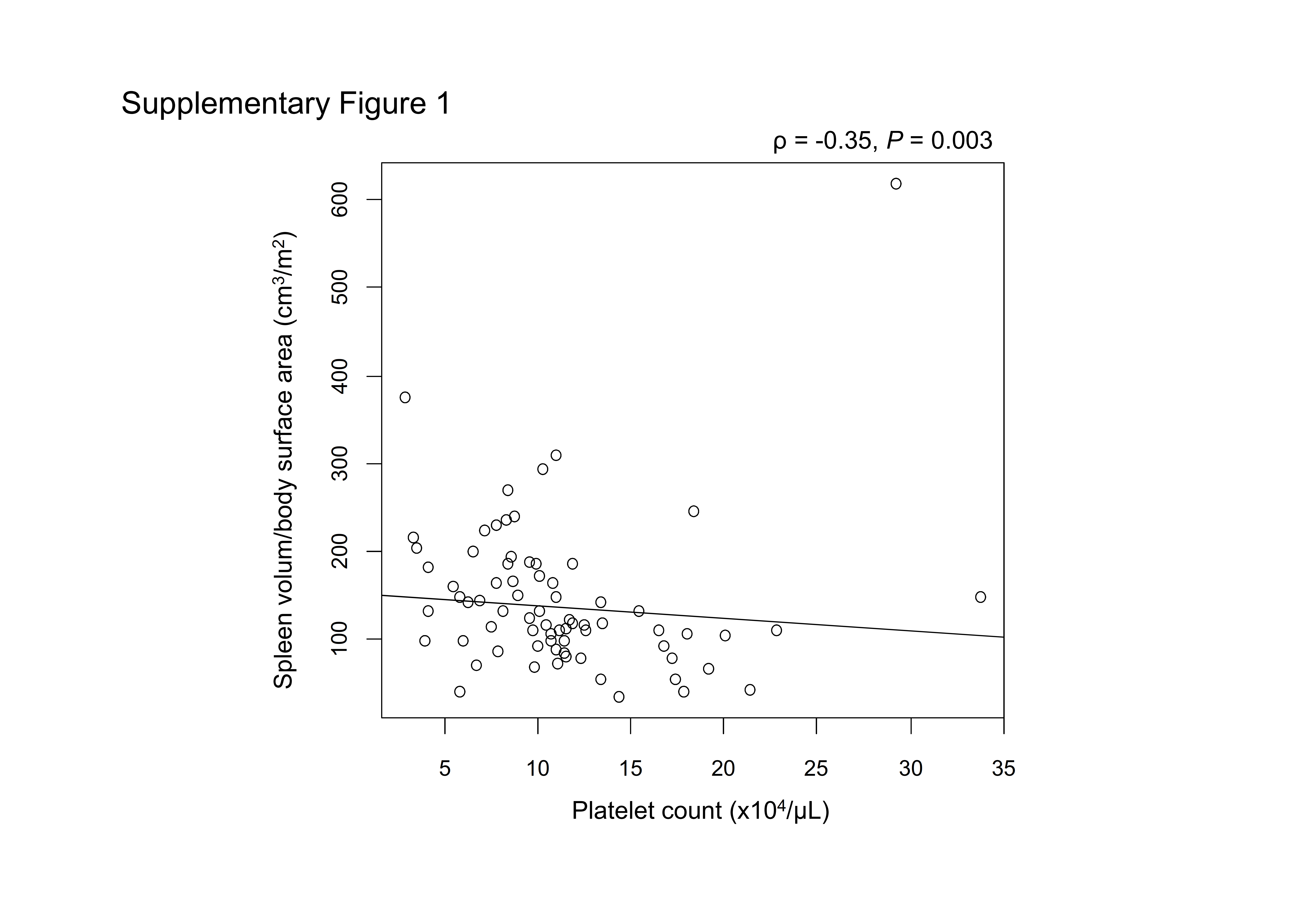

Supplement: S1 Fig — (TIF) [file pone.0231836.s004.tif]

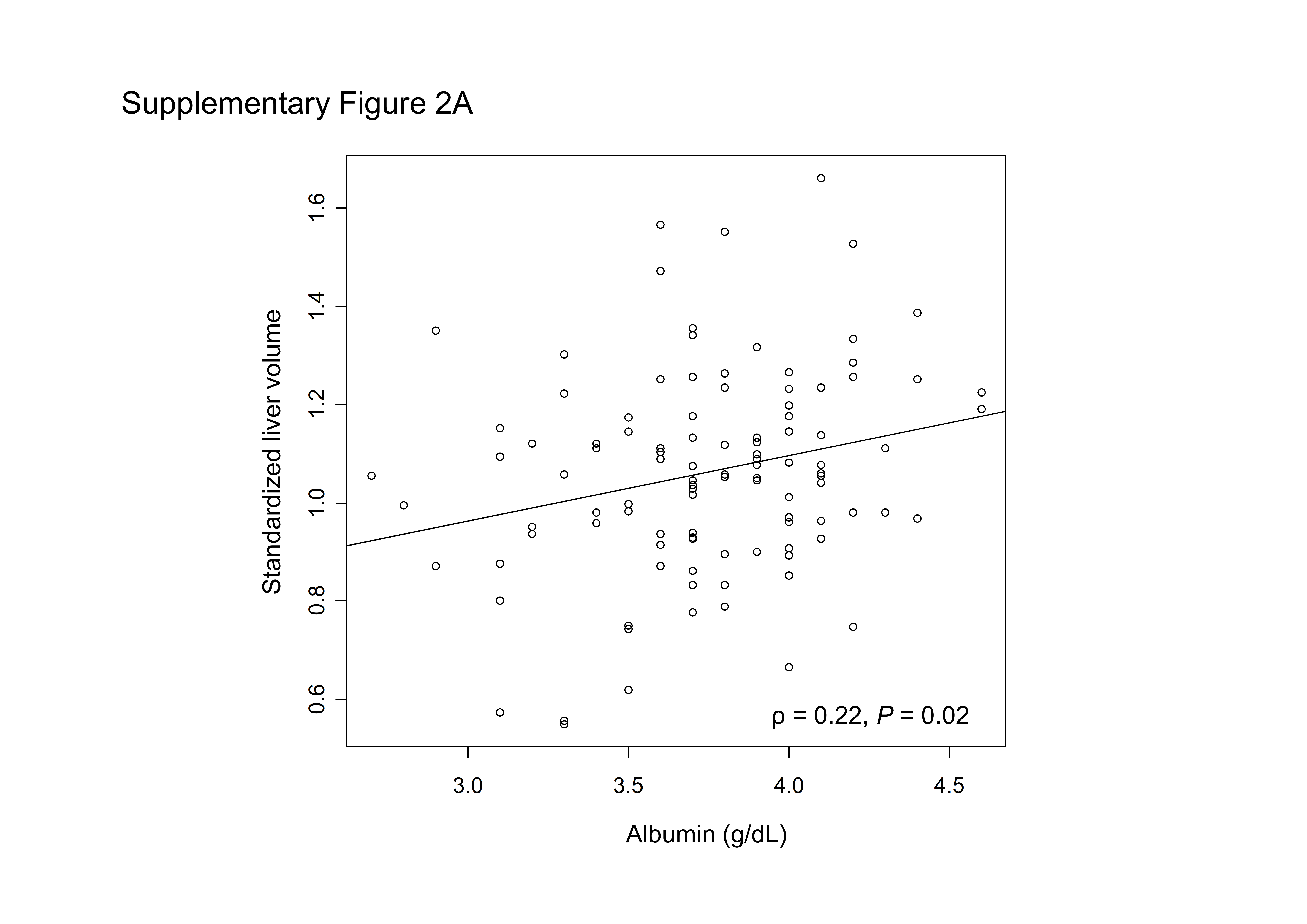

Supplement: S2 Fig — (A) Correlation between albumin level and liver volume at baseline. (B) Correlation between FIB-4 and liver volume at baseline. FIB-4, Fibrosis-4. (TIF) [file pone.0231836.s005.tif]

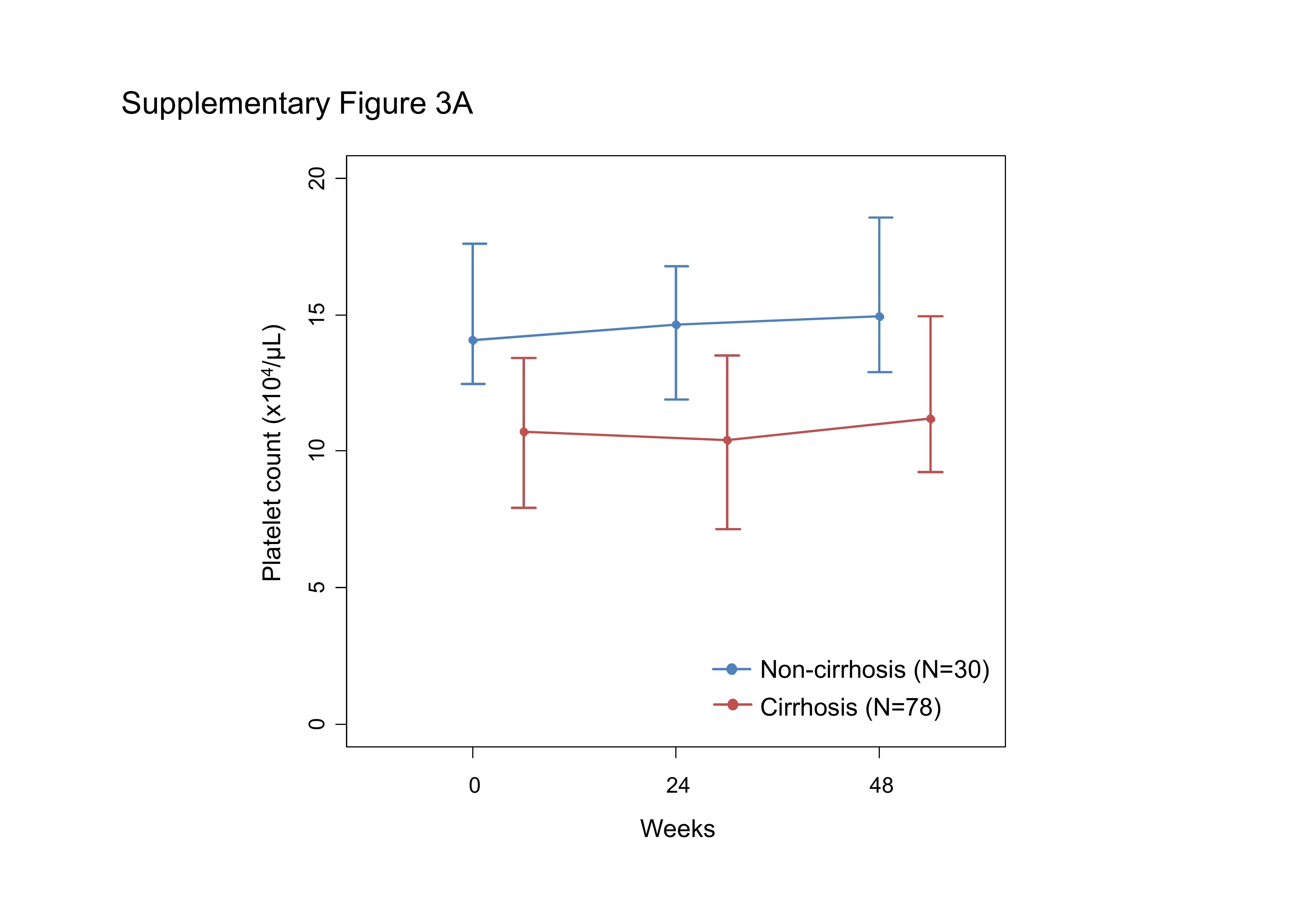

Supplement: S3 Fig — Points and error bars indicate median values and 25th to 75th percentiles, respectively. (TIF) [file pone.0231836.s006.tif]

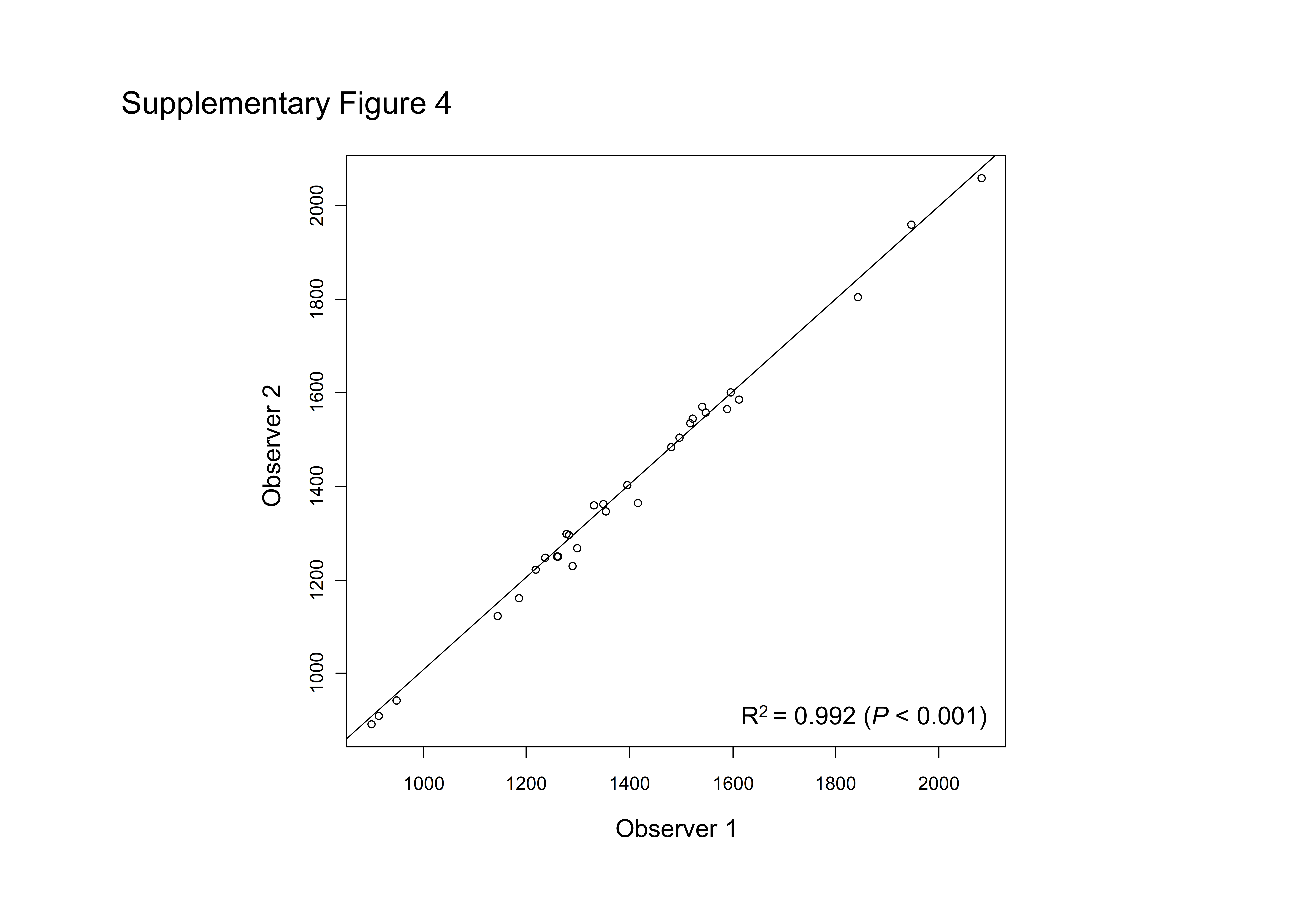

Supplement: S4 Fig — There was a correlation of 0.99 between values measured by 2 observers. (TIF) [file pone.0231836.s007.tif]

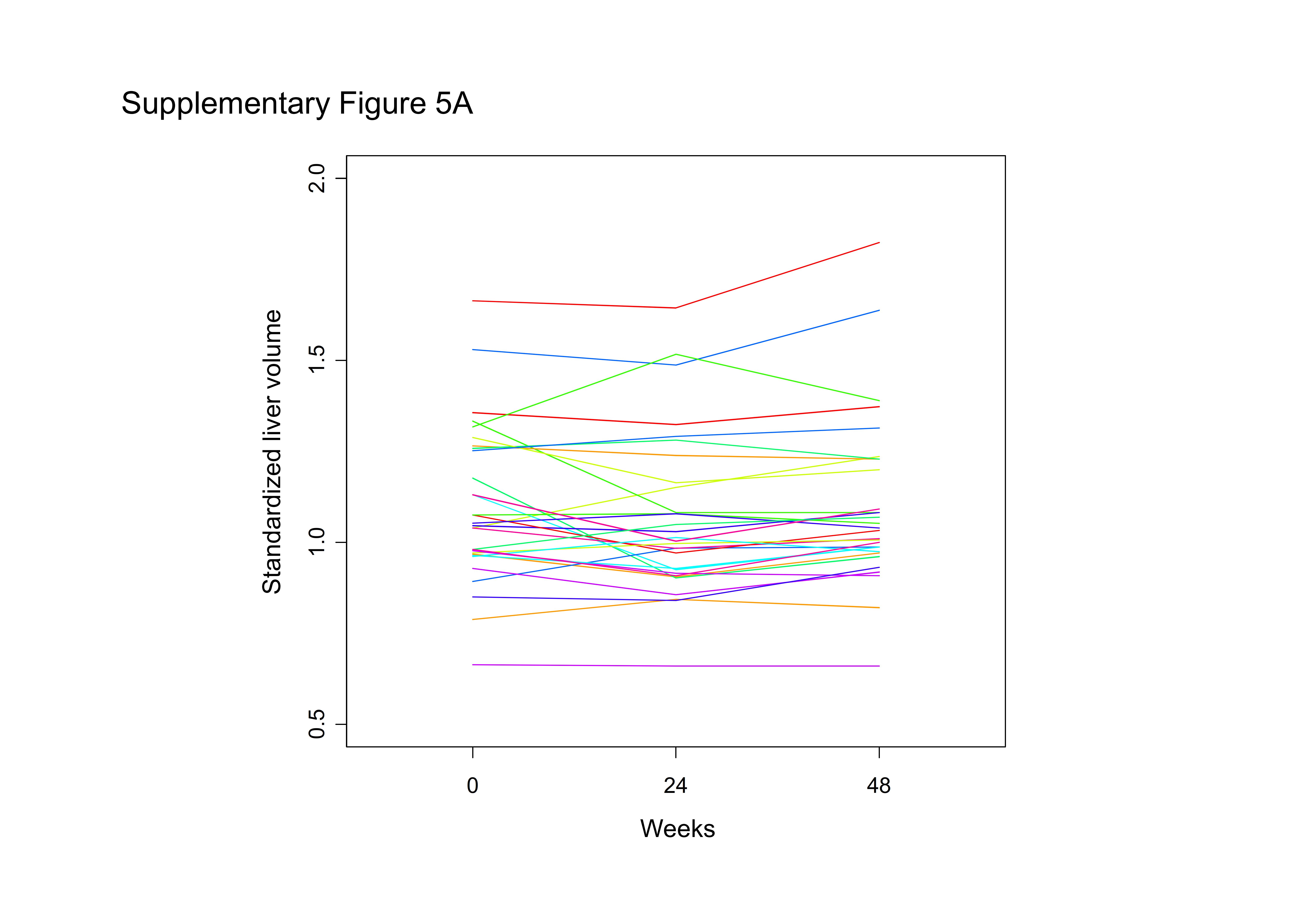

Supplement: S5 Fig — Each line indicates a unique patient. (TIF) [file pone.0231836.s008.tif]
